# Cost-Effectiveness of a New Internet-Based Monitoring Tool for Neonatal Post-Discharge Home Care

**DOI:** 10.2196/jmir.2361

**Published:** 2013-02-18

**Authors:** Valentina Isetta, Carme Lopez-Agustina, Esther Lopez-Bernal, Maribel Amat, Montserrat Vila, Carme Valls, Daniel Navajas, Ramon Farre

**Affiliations:** ^1^Unit of Biophysics and BioengineeringFaculty of MedicineUniversity of BarcelonaBarcelonaSpain; ^2^CIBER de Enfermedades Respiratorias (CIBERES)BunyolaSpain; ^3^Neonatal UnitPediatrics DepartmentHospital de la Santa Creu i de Sant PauBarcelonaSpain; ^4^Institut de Bioenginyeria de Catalunya (IBEC)BarcelonaSpain; ^5^Institut d’Investigacions Biomediques August Pi Sunyer (IDIBAPS)BarcelonaSpain

**Keywords:** Telemedicine, telenursing, Internet, neonatology, cost-effectiveness

## Abstract

**Background:**

The application of information and communication technologies in nursing care is becoming more widespread, but few applications have been reported in neonatal care. A close monitoring of newborns within the first weeks of life is crucial to evaluating correct feeding, growth, and health status. Conventional hospital-based postdischarge monitoring could be improved in terms of costs and clinical effectiveness by using a telemedicine approach.

**Objective:**

To evaluate the cost-effectiveness of a new Internet-based system for monitoring low-risk newborns after discharge compared to the standard hospital-based follow-up, with specific attention to prevention of emergency department (ED) visits in the first month of life.

**Methods:**

We performed a retrospective cohort study of two low-risk newborn patient groups. One group, born between January 1, 2011, and June 30, 2011, received the standard hospital-based follow-up visit within 48 hours after discharge. After implementing an Internet-based monitoring system, another group, born between July 19, 2011, and January 19, 2012, received their follow-up with this system.

**Results:**

A total of 18 (15.8%) out of 114 newborns who received the standard hospital-based follow-up had an ED visit in the first month of life compared with 5 (5.6%; *P*=.026) out of 90 infants who were monitored by the Internet-based system. The cost of the hospital-based follow-up was 182.1€ per patient, compared with 86.1€ for the Internet-based follow-up.

**Conclusion:**

Our Internet-based monitoring approach proved to be both more effective and less costly than the conventional hospital-based follow-up, particularly through reducing subsequent ED visits.

## Introduction

The economic policies of Western countries are increasingly pushing toward reductions in health care costs, especially through the avoidance of unplanned hospital-based services. In this context, telemedicine is progressively becoming more widely used as a valuable technique for delivering nursing care, particularly in a patient’s home, due to its capacity to provide efficient, long-distance service. Besides reducing the costs and problems related to patients’ need to travel to health facilities, telemedicine enhances patients’ involvement in their own care and reinforces the nurse-patient relationship [1].

One nursing care area in which telemedicine applications are still little seen is neonatology. Monitoring newborns in the first weeks of life is critical for assessing adequate feeding and weight gain and identifying alterations such as hyperbilirubinemia [2]. Since postpartum hospitalization has been gradually shortening in length over the past years, there is more chance of newborns’ parents/caregivers failing to recognize conditions requiring intervention, such as jaundice, dehydration, cardiac lesions, and serious infections [3-5]. Moreover, shorter hospital stays leave less time for the parental education and training that traditionally follow a baby’s birth [2]. This problem may be aggravated by an inconsistent or poorly scheduled follow-up after hospital discharge. In fact, some recent data suggest that postdischarge care for newborns may actually have worsened [6,7]. One major consequence of this is unplanned use of health care services, including emergency department (ED) visits and hospital readmissions.

Therefore, it would be of great use to have a support tool that would provide a continuum of care during the first weeks of a newborn’s life after going home [8]. Such a tool would facilitate contact and information-sharing between parents and specialized nurses, thereby enhancing parents’ confidence and their involvement in their baby’s care [9,10]. It would also be cost-effective, as it would relieve the pressure on the health care system caused by unplanned hospital-based care.

The aim of this study was to implement and evaluate an innovative postdischarge monitoring strategy for newborn patients involving a new Internet-based support system. This telemedicine tool (called “Babies at home”) includes a web application that provides educational information about neonatal care to new parents, as well as baby monitoring via a questionnaire that parents fill in periodically and an email service that offers easy communication between parents and nurses.

To assess the effectiveness and financial viability of this new neonatal telemedicine service, a cost-effectiveness analysis was carried out by comparing this tool with the traditional hospital-based postdischarge follow-up. We hypothesized that an Internet-based support system for monitoring newborn patients after discharge from nursery would improve care, be well accepted by parents, and reduce unplanned health care, particularly ED visits.

## Methods

**Figure 1:**
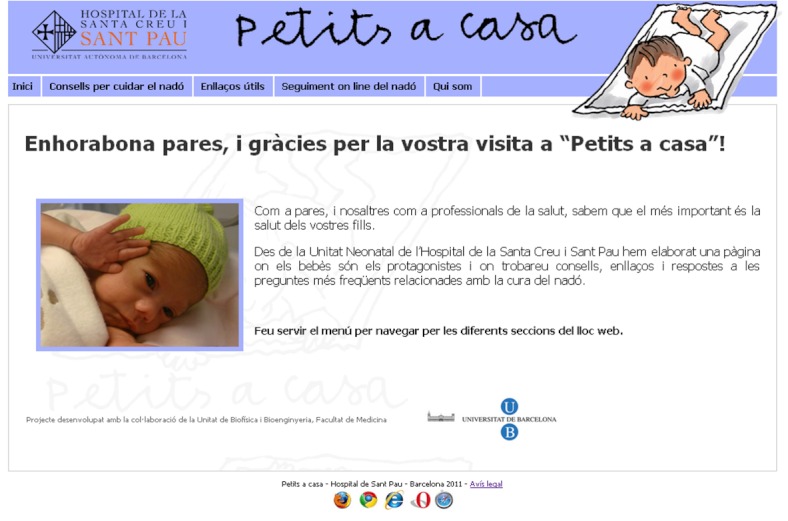
“Babies at home” home page screenshot.

The “Babies at home” web monitoring system (see Figure 1 and Multimedia Appendix 1 for translation to English) was designed, put into clinical service, and evaluated in a collaborative study involving the Neonatal Care Department of the Hospital de la Santa Creu i Sant Pau (HSP) of Barcelona and the Biophysics and Bioengineering Unit of the University of Barcelona. It has been in clinical use since July 2011 [11].

### Study Population

We conducted a retrospective cohort study on newborn patients born between January 1, 2011, and January 19, 2012, in the HSP delivery rooms. Patients included in the study were consecutive low-risk newborns, specifically (1) late preterm newborns, born between 35 and 37 weeks of gestation, (2) babies weighing between 2200g and 2500g, (3) babies weighing between 2500g and 3000g who were the first children and received only breastfeeding, (4) babies weighing over 3000g who were the first children and/or received only breastfeeding and suffered a weight reduction of over 7% after birth, and (5) babies whose home was more than 40 km away from the hospital. Parents who were unable to communicate in written Spanish or Catalan or had no Internet access at home were excluded from the study.

Our study was based on a before/after design, which included one group of patients from before the implementation of the Internet-based follow-up and another from afterward. One group of babies (control group), born between January 1 and June 30, 2011, received the standard hospital postdischarge follow-up, which consisted of a hospital visit within 48 hours of the newborn’s discharge. The other group (intervention group), born between July 19, 2011, and January 19, 2012, was monitored by the new Internet-based tool.

### Internet-Based Monitoring System “Babies at home”

We developed the Internet-based monitoring tool “Babies at home” as a dynamic server-side website based on PHP language and MySQL database on a Linux/Apache server. All the development phases, from the structural design to the content formulation, evolved in close collaboration with the medical/nursing staff. The application comprises three main areas:

1. Free-access Area: open platform where all parents can find extensive high-quality information about baby care and useful links to breastfeeding and neonatal nursing association websites (see Multimedia Appendix 2).
2. Parents’ Area: restricted area where only registered parents can access after authentication (see Multimedia Appendix 3). They are asked to answer twice a week a questionnaire from the neonatal nurses about the baby’s conditions (weight, feeding, sleeping, body temperature, skin color, etc.), thereby covering the essential topics that nurses usually assess during hospital visits. All data are sent to a MySQL relational database stored in the secure environment of the hospital server. Parents have visual feedback of their baby’s weight trend, plotted and continuously updated on the basis of the answers on the periodic questionnaires. Another important feature is the option of exchanging email messages with the nurses, making it possible to raise doubts and answer questions about baby care.
3. Staff Area: by logging in, neonatal nurses and pediatricians can access their special area, where they can monitor parents’ answers to the questionnaires retrieved from the database, shown in dynamic Flash charts (see Multimedia Appendix 4). After evaluating the baby’s data, nurses can write a message directly from the website to the parents to provide advice and comments about the newborn’s care.

Once the patients’ eligibility was established, the parents of the children participating in the study signed an informed consent form including a privacy protection statement, which was written with the endorsement of the hospital regulatory department. Before leaving the hospital, the latter were taught how to use the website and supplied with appropriate information, both general and specific, about baby care and also reassured about their capacity and commitment to take care of their baby at home. The neonatal nursing staff was in charge of both this initial training and the monitoring of the baby, undertaken by periodically checking parents’ answers to the online questionnaire. In the event of any discrepancy in any parameter, nurses were able to contact the family by email or phone to check the newborn’s conditions and address any possible problems in a suitable manner. Parents were also able to directly contact the nursing staff by email to ask questions and clarify any doubts about neonatal care. The baby’s condition continued to be monitored until they achieved an appropriate weight and condition. At the end of the monitoring period, the parents were kindly invited to answer a final online survey to assess their level of satisfaction with the web service (see Multimedia Appendix 5). The survey consisted of 9 statements about the usefulness of the website contents and functions, and the possible answers were distributed on a Likert scale from 0 (“I strongly disagree”) to 5 (“I strongly agree”).

### Outcomes

The main goal of our evaluation study was to assess the effectiveness of the new postdischarge Internet-based monitoring tool, which was evaluated in terms of the ED service used by the study population in the first month after discharge, before and after the implementation of the Internet-based follow-up. To this end, the parents were asked to preferentially use the ED of our hospital if an emergency visit was required and to report any visit to another ED. Accordingly, we performed a cost-effectiveness analysis from a social perspective. Our main outcome measures were (1) the follow-up cost per patient, (2) the rate of newborns who did not require an ED visit in the first month after birth, either because they did not need it or because the nursing support received via the Internet-based monitoring allowed them to avoid it, and (3) the incremental cost-effectiveness ratio (ICER) of the Internet-based follow-up compared to usual care. Another outcome of interest was the rate of ED visits. The ICER is commonly used in health economics and is a standard measure for cost-effectiveness analysis [12]. It represents a measure of the additional cost per unit of health gain, which in our case is one ED visit required by a newborn within the first month of life. The ICER is computed as follows: ICER=(C_IF_-C_HF_)/(E_IF_-E_HF_), where C_IF_ is the cost of the Internet-based follow-up strategy, C_HF_ is the cost of the hospital-based follow-up strategy, E_IF_ is the effectiveness of the Internet-based follow-up strategy and E_HF_ is the effectiveness of the hospital-based follow-up strategy. The effectiveness values were measured in terms of the avoidance of hospital-based care services.

### Cost Measurements

We considered both direct and indirect costs for the cost-effectiveness analysis (Table 1). Immaterial costs were not taken into account. Direct health costs were associated with the use of health care resources and were classified as ED visits, hospital visits, and nursing personnel costs related to the web monitoring. Information about the costs of ED and hospital visits and the nurses’ hourly salary were provided by the administrative department of the hospital (which participates in the Catalonian Public Health Service). The average time taken to train newborns’ families before leaving the hospital (10 minutes per family) and to perform the monitoring with the Internet-based tool (5 minutes per baby per day) was determined through interviews with the nursing staff in charge of it. Direct nonhealth costs corresponded to the travel expenses incurred by newborns’ families to go to the hospital for ED or hospital visits. We also took into account indirect costs, such as opportunity costs related to parents’ missed work time due to those visits. We considered that just one parent came to the hospital with the child. The most frequent transportation mode for parents to come to the hospital for a visit (in the city center) was by car or taxi. Average transport costs were estimated, considering that the study population lived within the Barcelona district. These travel costs were calculated as an average between the taxi fare paid by a family living near the hospital (5-10€ approximately) and one paid ride from the city surroundings (45-50€ approximately). The cost of work time missed by the parents due to ED or hospital visits was calculated considering the average annual wage in Spain [13] and a regular weekly work time of 40 hours. We estimated an average of 3 hours lost for an ambulatory visit and 5 hours for an ED visit, considering the total sum of waiting, visit, and travel times.

**Table 1:** Direct and indirect costs included in the analysis (cost sources and ranges considered in the study are indicated).

		Cost, €	Range, %	Source
**Direct Health Costs**				
	ED visit	127.2	±75	HSP
	Hospital visit	85.4	±75	HSP
	Nurse’s hourly salary	33.0	±75	HSP
**Direct Nonhealth Costs**				
	Transport to hospital	30.0	±75	Assumption
**Indirect Costs**				
	Missed work hour	15.0	±75	Assumption

### Statistical Analysis

Comparison of ED visit rates between patients who were monitored by the Internet-based system and those who received the standard hospital-based follow-up was made using Fisher’s exact test.

## Results

### Study Groups

From January 1, 2011, to January 19, 2012, 931 newborns were discharged from the HSP nursery. Of these, 230 met the criteria to be included in the study. The study population was divided into two groups: (1) the postdischarge follow-up for 114 newborns consisted of a hospital visit within the 48 hours after discharge from January 1 to June 31, 2011 (control group), and (2) for 116 infants this was performed using the Internet-based system “Babies at home” from July 19, 2011, to January 19, 2012 (intervention group). Out of 116 families included in the program, 90 (77.6%) participated actively until the end of their newborns’ monitoring period and they were considered for the study. While each newborn from the control group received the standard hospital-based follow-up, in the intervention group, 32 infants needed a hospital visit due to neonatal or maternal pathology or because the nursing staff in charge of the Internet-based monitoring considered it appropriate.

### Internet-Based Monitoring System and Parents’ Satisfaction

Nursing staff received 382 answers to the online questionnaire (an average of 4.7 answers per patient) and 90 email consultations from parents (an average of 1 email per patient). Forty-eight percent of parents (N=43) answered the final satisfaction survey. Globally, they professed to be generally satisfied with the web service, showing a level of agreement of 4.3 ± 0.9 (mean ± SD) to the first satisfaction survey statement, equivalent to an overall positive evaluation of the helpfulness of the “Babies at home” website (Table 2).

**Table 2:** Results of patients’ satisfaction survey (mean ± SD) where 0 means “I strongly disagree” and 5 means “I strongly agree”.

Survey statement	Answer (mean ± SD)
1. In general the Web service “Babies at home" was helpful.	4.3 ± 0.9
2. The available information helped me take care of the baby.	4.3 ± 0.8
3. The information available on the website could clarify my doubts.	4.1 ± 0.9
4. The email service with nurses available on the website was useful.	4.6 ± 0.9
5. The files and recommended links were useful.	4.0 ± 1.0
6. The nurse’s answers to my questions were useful.	4.7 ± 0.9
7. The use of the website avoided visits to the primary care center.	31 (yes) – 72%
8. The use of the website avoided visits to the emergency department.	16 (yes) – 37%
9. I would recommend this website.	41 (yes) – 95%

### ED Visits

According to the instructions given to parents, our ED was the only one visited during the study. Eighteen (15.8%) of the 114 newborns who received the standard hospital-based follow-up returned to the hospital’s ED in the first month after birth compared with 5 (5.6%) of the group monitored by the Internet-based tool (*P*=.026).

### Cost-Effectiveness Analysis

Considering the first month after discharge, 94.4% of the patients who received the Internet-based follow-up had no ED visits, compared with 84.2% of the control group patients. Our analysis revealed that the cost of the Internet-based follow-up per patient was 86.1€, while the hospital-based follow-up cost per patient was 182.1€ (Table 3). Therefore, the Internet-based follow-up strategy is said to be dominant because it is both less costly and more effective. The ICER of the Internet-based follow-up strategy compared with the standard hospital visit was -941.2€, which implies that society will save 941.2€ for every additional infant who does not have an ED visit in the first month of life.

The programming costs of the Internet-based tool corresponded to 1.5 person/month, ie, 7,500€ in a programmer’s salary, including all taxes. Since the hospital already had the required computer and communication equipment, including a secure server, we only considered the cost of the programmer’s salary for the tool development. With a savings of 96.0€ per patient follow-up, the Internet-based tool programming recovered its cost after 79 patients’ follow-up—a figure that was attained within 6 months of the implementation of the new monitoring strategy.

**Figure 2:**
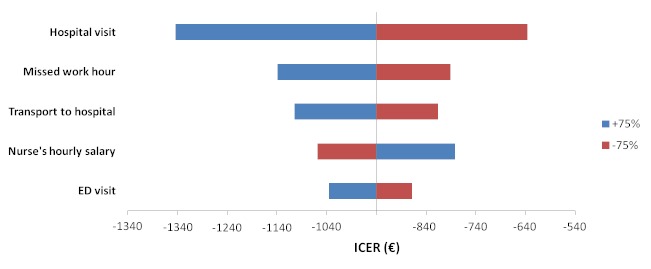
Tornado diagram showing the impact of cost parameters on ICER.

One-way sensitivity analyses for ED visit cost, hospital visit cost, nurses’ hourly salary, cost of families travelling to hospital and parents’ missed work time revealed, within a plausible range of selection (±75%), that the Internet-based follow-up was still superior to the standard hospital-based follow-up. The tornado diagram in Figure 2 shows the impact of each cost parameters on ICER.

Varying the cost values simultaneously in two different cost scenarios, the Internet-based strategy was still dominant in comparison to the standard one (Table 4).

**Table 3:** Costs per infant and ICER of Internet-based follow-up for prevention of ED visits in the first month of life.

Strategy	Cost	Incremental cost	Effectiveness	Incremental effectiveness	ICER
Internet-based follow-up	86.1€	96.0€	0.944	-0.102	-941.2€
Hospital Visit	182.1€		0.842		

**Table 4:** Sensitivity analyses for two additional cost scenarios.

Costs (€)	Scenario +75%	Base case	Scenario -75%
Hospital visit	149.5	85.4	37.4
Missed work hour	26.3	15.0	6.6
Transport to hospital	52.5	30.0	13.1
Nurse’s hourly salary	57.8	33.0	14.4
ED visit cost	222.6	127.2	55.7
Cost per patient	150.7-318.6	88.1-182.1	37.7-79.7
ICER	–1646.3	–941.2	–411.6

## Discussion

### Main Results

To our knowledge, this study is the first financial viability assessment of a telemedicine intervention in the neonatal postdischarge home care field. The results of the cost-effectiveness assessment provide support to the introduction of telemedicine services into routine clinical practice [14]. Specifically, the use of an Internet-based follow-up system to monitor low-risk newborns in the first month of life is both less costly and more effective than the usual hospital-based follow-up. This new monitoring approach resulted in a significant reduction in the subsequent use of hospital-based resources, such as ED visits, after discharge and a high level of parental satisfaction with the service. This reduction in ED visits can be considered not only a clear cost reduction for the health care provider but also an improvement in the newborn’s clinical outcome after early discharge [15].

Besides providing high-quality educational contents about neonatal care to parents, “Babies at home” offers nursing staff a valuable and easy procedure for the home monitoring of newborns, as well as fast long-distance communication with families. An interactive website is a very convenient method, due to the wide availability of Internet-connected devices among health care consumers, especially in the homes of young families. Moreover, since usability and structural simplicity were crucial to the development of the application, the training required by nurses and parents is minimal. Concerns about security and data confidentiality have been minimized, as this application was easily incorporated into the secure environment of the hospital server, in compliance with all the applicable legal regulations.

### Other Outcomes

Our analysis focused on assessing the clinical effectiveness of our Internet-based monitoring approach during the first month of the newborn’s life. Extending the assessed period to 2 months after birth, which is considered a postnatal phase, we found that 29 infants (25.4%) who received the standard hospital-based follow-up had an ED visit, compared with 7 (7.8%) of those monitored by the new Internet-based system (*P*=.0014). Since the Internet-based monitoring period was generally no longer than 1 month, we may conclude that our approach also had a positive impact on families’ education and empowerment with respect to their babies’ daily care and that this translated into a more pronounced decrease in the use of the ED.

Furthermore, this enhancement of parental education also had a positive effect on the continuance of breastfeeding, which is essential for babies’ health and growth [16]. It has been widely demonstrated that the breastfeeding rate generally decreases by 10-20% within the first month of a baby’s discharge [17,18]. Remarkably, the mothers included in the “Babies at home” program maintained the same breastfeeding rate after 1 month as at discharge (60%), with no reduction at all. This success in avoiding a decrease in breastfeeding could be attributed both to the access the mothers had to the informative and educational items available on the website and to the fluid contact with nurses during the monitoring period.

### Comparison With Previous Studies

In the last decade, the telemedicine concept has been expanded to nursing care because of its capacity to provide efficient long-distance health care. Most nurses recognize the contribution that information and communication technology, particularly the Internet, can make to both their practice and patients’ understanding of their own health and care [19]. There are several examples of telenursing applications in the literature. Internet-based tools for chronic disease management, such as dyspnea in COPD patients [20], for educational intervention, such as Web-assisted tobacco control [21], or to support nurse-led triaging [22], are recent examples of successful telenursing applications using the Web.

In the neonatal care field, several telemedicine applications have been previously developed, particularly to bring real-time diagnoses to neonatal facilities without in-house trained specialists. These include the remote evaluation of digital images for retinopathy in prematurity screening [23,24], interventions for deaf or hard-of-hearing infants [25], the long-distance interpretation of echocardiograms [26], and neonatal teleconsultations in general [27].

Nevertheless, very few researchers have developed Internet-based tools for supporting families in the care of their newborns during the first days of life. One study described a program in which nurses provided updates to family members of Neonatal Intensive Care Unit (NICU) patients on the Internet [28]. The authors reported significant improvements in family satisfaction with NICU in the in-patient care of babies with very low birth weights and pointed out the need to extend this service to the postdischarge period. In another study, parents viewed real-time video images of their hospitalized newborns via an Internet browser or 3G cell phone [29]. Although no significant impact was found in terms of newborns’ length of hospital stay, this virtual visiting was well accepted by families, suggesting the advisability of evaluating its role in improving postdischarge transition care.

### Other Potential Clinical Applications

Thanks to its simplicity and versatility, our Internet-based neonatal monitoring system could be easily adapted to a wider range of application. First, its application could be useful in developing countries, where remote and poor places need simple and cheap technological interventions to give access to beneficial health services to those most in need [30]. Also, our tool could be effectively integrated into programs of neonatal postdischarge home assistance, which provide in-home support from clinical nursing specialists after infants’ discharge [31] but are hampered by clinical and geographical constraints that render this service inaccessible to many families that could otherwise benefit from it. An Internet-based monitoring tool could effectively overcome such limitations and give more families access to such programs.

### Limitations

This was a retrospective study. Our Internet-based follow-up system was implemented as a possibly more efficient and cheaper monitoring strategy. The markedly improved efficiency of our new system was evident right from the start. Treating some patients with an efficient system, while randomizing others to an inefficient system, precluded any randomized controlled trial. Moreover, it was logistically impossible within the hospital guidelines and policies to run both postdischarge systems simultaneously.

It should be pointed out that the control and intervention groups were analyzed in two different periods of the year. However, the rate of ED visits should have not been affected by any seasonal bias taking into account that each of the two time windows (January-July and August-January) shared the same number of winter months. Actually, the main season-related causes of ED visits and hospitalizations in infants up to 1-month old are viral infections, such as respiratory syncytial and flu [32], which exhibit a well-known incidence distributed through the winter months [33].

One advantage of our study was the use of real data of clinical costs and effectiveness in terms of ED use. It is worth noting that, although these cost values were measured for a public university hospital in a big Spanish city, Barcelona, the positive results obtained in this study could easily be translated to other public or private health systems providing postnatal care, taking into account the results of the sensitivity analyses confirming the cost-effectiveness of the new telemedicine strategy in different cost scenarios.

### Future Directions

“Babies at home” can be gradually expanded and optimized. To facilitate the assessment of some clinical parameters, such as a newborn’s skin color, navel care, and correct breastfeeding, the web tool can be incorporated into a parent-nurse videoconference communication system. Also, the tool can be completely integrated into the hospital EHR system, so that the nursing staff in charge of web monitoring can easily access the patient’s record, which would be automatically updated with the main clinical events and issues assessed during the telemonitoring.

### Conclusion

Using the rate of ED visits and the costs for society, the cost of the Internet-based follow-up was shown to be much lower than that of the conventional hospital-based follow-up. Additionally, ED visits in the first month of patients’ life decreased with the use of the Internet-based monitoring system. This telemedicine follow-up strategy proved absolute dominance (both more clinically effective and less costly) over the standard follow-up based on hospital visits.

## Multimedia Appendix 1

Translation for “Babies at home” home page screenshot.

## Multimedia Appendix 2

Translation for “Tips for baby care” page in the free access area of “Babies at home” website.

## Multimedia Appendix 3

Translation for "Parents’ area" access page screenshot.

## Multimedia Appendix 4

Translation for "Visualization of some of the parents’ answers to the periodic questionnaire by dynamic Flash charts".

## Multimedia Appendix 5

Translation of Final online satisfaction survey.

## Abbreviations

ED: emergency department
HSP: Hospital de la Santa Creu i Sant Pau
ICER: incremental cost-effectiveness ratio
NICU: Neonatal Intensive Care Unit
