# Supplementary material for: Cost-Effectiveness of a New Internet-Based Monitoring Tool for Neonatal Post-Discharge Home Care
Source: J Med Internet Res. 2013 Feb 18;15(2):e38. doi: 10.2196/jmir.2361 (PMC3636285; doi:10.2196/jmir.2361)

Multimedia Appendix 1: Translation for “Babies at home” home page screenshot.

Translation from original version in Catalan: *Header:* “Babies at home”. *Menu-bar:* “Home”, “Tips for baby care”, “Useful links”, “Online baby follow-up”, “About us”. *Content:* “Congratulations, parents, and thank you for your visit to ‘Babies at home’! You as parents, and we as health professionals, know that the most important thing is the health of your children. In the Neonatal Unit at the Hospital de la Santa Creu i Sant Pau we have created a page where the babies are the stars and you will find tips, links and answers to frequently asked questions regarding baby care. Use the menu to navigate to different sections of the website.”, “Project developed with the collaboration of the Unit of Biophysics and Bioengineering, Faculty of Medicine, University of Barcelona.”

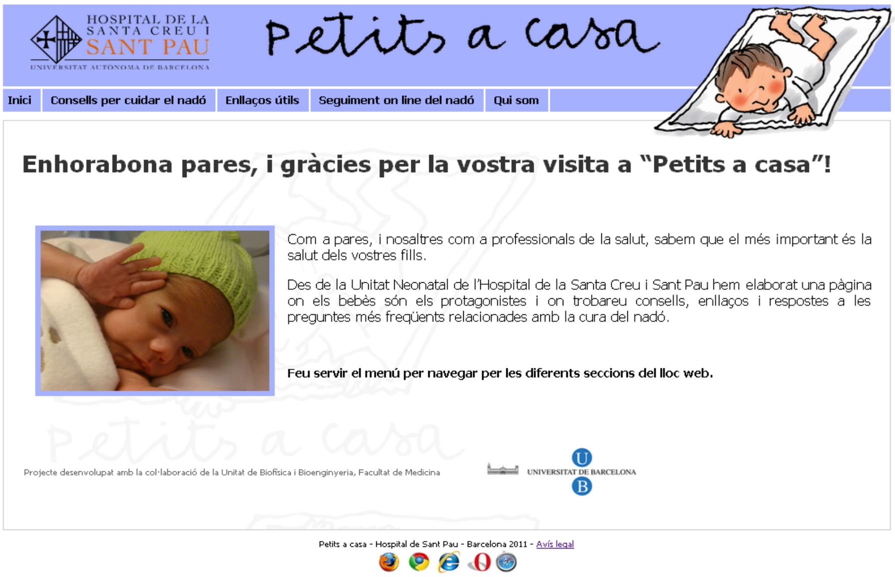

Supplement: Supplementary file 1 [file jmir_v15i2e38_app1.pdf]
