# Supplementary material for: Cost-Effectiveness of a New Internet-Based Monitoring Tool for Neonatal Post-Discharge Home Care
Source: J Med Internet Res. 2013 Feb 18;15(2):e38. doi: 10.2196/jmir.2361 (PMC3636285; doi:10.2196/jmir.2361)

Multimedia Appendix 2: Translation for “Tips for baby care” page in the free access area of “Babies at home” website.

Translation from original version in Catalan and Spanish: *Header:* “Babies at home”. *Menu-bar:* “Home”, “Tips for baby care”, “Useful links”, “Online baby follow-up”, “About us”. *Content:* “Tips for baby care”. “Breastfeeding: Why breastfeeding?; When breastfeeding?; How should you do it?; Most frequent problems; Lactation of premature babies; Lactation of twins; Milk extraction and conservation; Toxic substances which pass to breast milk; References; Support groups, videos and interesting websites”. “Artificial lactation: Feeding bottle preparation”. “Sensory stimulation: Recommended activities in the first month of life”.

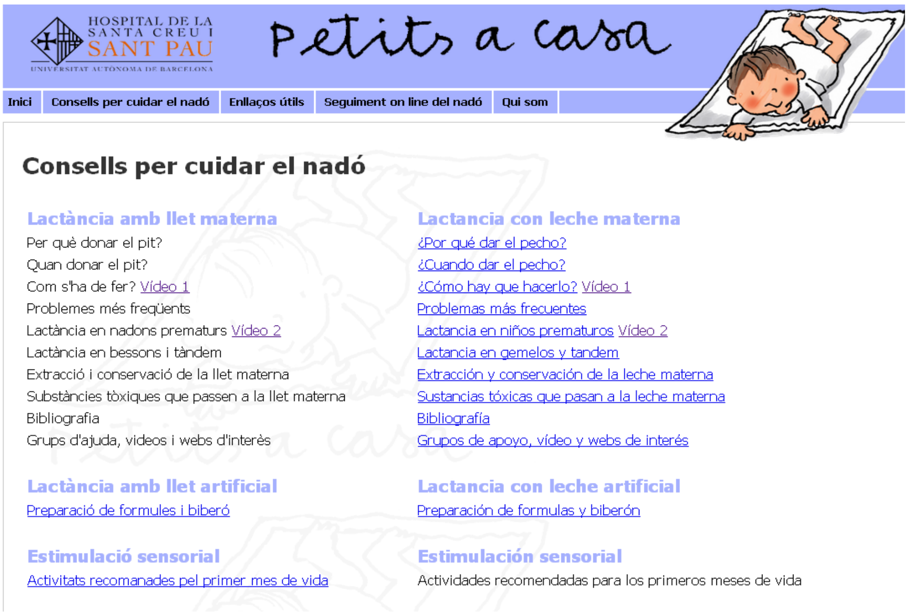

Supplement: Supplementary file 2 [file jmir_v15i2e38_app2.pdf]
