# Supplementary material for: Cost-Effectiveness of a New Internet-Based Monitoring Tool for Neonatal Post-Discharge Home Care
Source: J Med Internet Res. 2013 Feb 18;15(2):e38. doi: 10.2196/jmir.2361 (PMC3636285; doi:10.2196/jmir.2361)

Multimedia Appendix 3: Translation for "Parents' area" access page screenshot.

Translation from original version in Catalan: *Header:* "Babies at home". *Menu-bar:* "Home", "Tips for baby care", "Useful links", "Online baby follow-up", "About us". *Content: "*Online baby follow-up. Access to this section is exclusively for parents registered to the online baby follow-up service.", "Login. Your e-mail address. Your password. Enter.", "Forgot your password?", "Healthcare professionals".

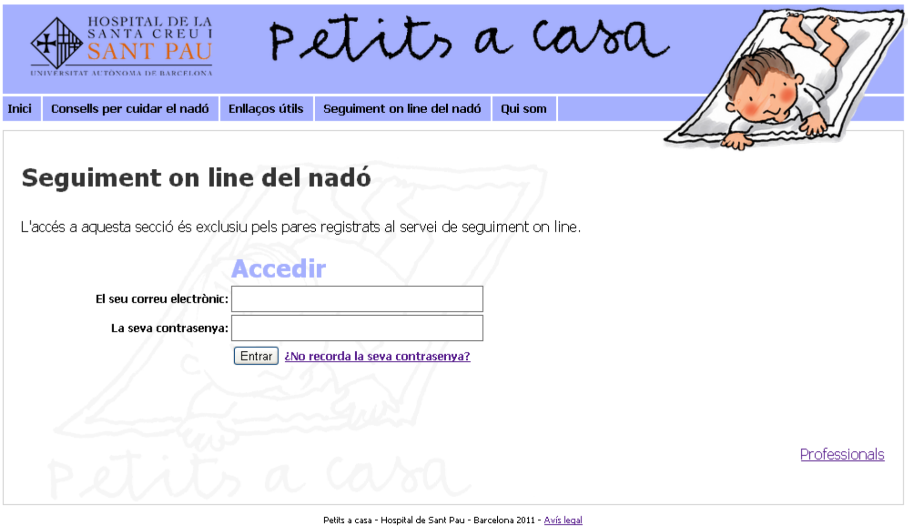

Supplement: Supplementary file 3 [file jmir_v15i2e38_app3.pdf]
