# Supplementary material for: Cost-Effectiveness of a New Internet-Based Monitoring Tool for Neonatal Post-Discharge Home Care
Source: J Med Internet Res. 2013 Feb 18;15(2):e38. doi: 10.2196/jmir.2361 (PMC3636285; doi:10.2196/jmir.2361)

Multimedia Appendix 4: Translation for "Visualization of some of the parents’ answers to the periodic questionnaire by dynamic Flash charts".

Translation from original version in Catalan: *Header:* “Babies at home. Online baby follow-up”. *Menu-bar:* “Baby record”, “Deregister a baby”, “Register a baby”, “Log out”. *Title:* ”Data of baby number…”. *Graphs:* “Weight”, “Feeding”, “Intakes per day”.

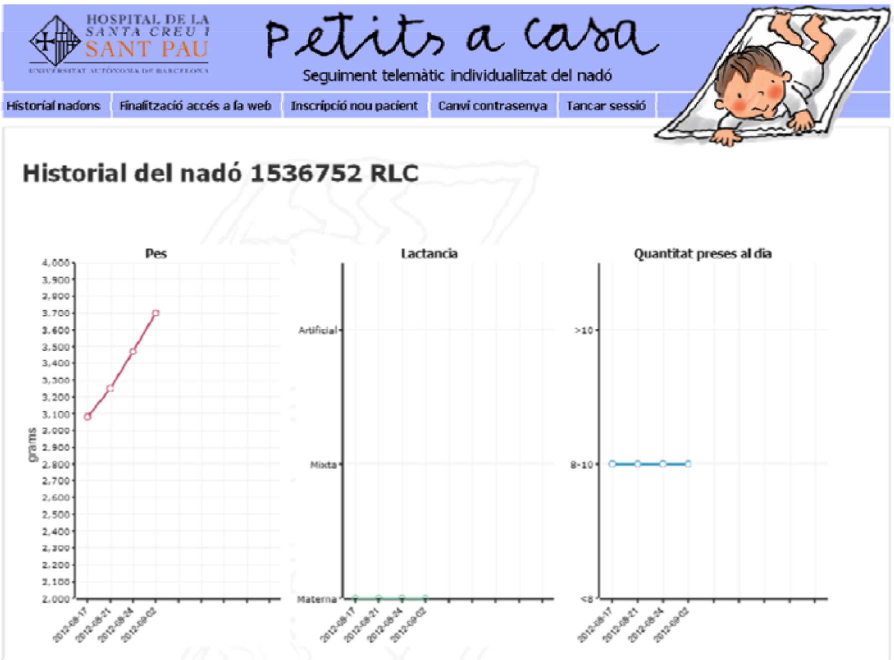

Supplement: Supplementary file 4 [file jmir_v15i2e38_app4.pdf]
