# Supplementary material for: Cost-Effectiveness of a New Internet-Based Monitoring Tool for Neonatal Post-Discharge Home Care
Source: J Med Internet Res. 2013 Feb 18;15(2):e38. doi: 10.2196/jmir.2361 (PMC3636285; doi:10.2196/jmir.2361)
Supplement: Supplementary file 5 [file jmir_v15i2e38_app5.pdf]

Multimedia Appendix 5: Translation of Final online satisfaction survey.

Translation from original version in Catalan: *Header:* “Babies at home. Online baby follow-up”. “Satisfaction survey. Please, choose an answer between 0 and 5 for each of the following questions (consider that 0 means “I strongly disagree” and 5 means “I strongly agree”)”.

1. “In general the Web service ‘Babies at home’ was helpful.”
2. “The available information helped me take care of the baby.”
3. “The information available on the website could clarify my doubts.”
4. “The e-mail service with nurses available on the website was useful.”
5. “The files and recommended links were useful.”
6. “The nurse’s answers to my questions were useful.”
7. “The use of the website avoided visits to the primary care center.”
8. “The use of the website avoided visits to the emergency department.”
9. “I would recommend this website.”
10. “Suggestions”

“Submit”

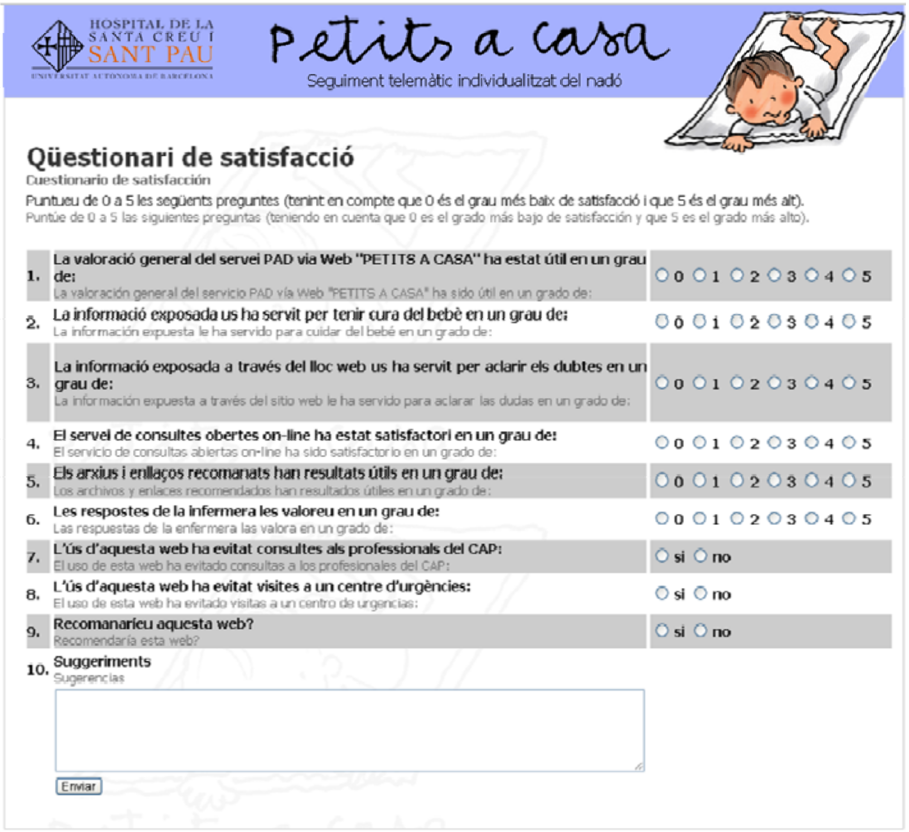
HOSPITAL DE LA SANTA CREU I SANT PAU
UNIVERSITAT AUTÒNOMA DE BARCELONA

Petits a casa

Seguiment telemàtic individualitzat del nadó

Qüestionari de satisfacció

Cuestionario de satisfacción

Puntueu de 0 a 5 les següents preguntes (tenint en compte que 0 és el grau més baix de satisfacció i que 5 és el grau més alt).
Puntúe de 0 a 5 las siguientes preguntas (teniendo en cuenta que 0 es el grado más bajo de satisfacción y que 5 es el grado más alto).

1.	La valoració general del servei PAD via Web "PETITS A CASA" ha estat útil en un grau de:<br>La valoración general del servicio PAD vía Web "PETITS A CASA" ha sido útil en un grado de:	0 1 2 3 4 5
2.	La informació exposada us ha servit per tenir cura del bebè en un grau de:<br>La información expuesta le ha servido para cuidar del bebé en un grado de:	0 1 2 3 4 5
3.	La informació exposada a través del lloc web us ha servit per aclarir els dubtes en un grau de:<br>La información expuesta a través del sitio web le ha servido para aclarar las dudas en un grado de:	0 1 2 3 4 5
4.	El servei de consultes obertes on-line ha estat satisfactori en un grau de:<br>El servicio de consultas abiertas on-line ha sido satisfactorio en un grado de:	0 1 2 3 4 5
5.	Els arxius i enllaços recomanats han resultats útils en un grau de:<br>Los archivos y enlaces recomendados han resultados útiles en un grado de:	0 1 2 3 4 5
6.	Les respostes de la infermera les valoreu en un grau de:<br>Las respuestas de la enfermera las valora en un grado de:	0 1 2 3 4 5
7.	L’ús d’aquesta web ha evitat consultes als professionals del CAP:<br>El uso de esta web ha evitado consultas a los profesionales del CAP:	si no
8.	L’ús d’aquesta web ha evitat visites a un centre d’urgències:<br>El uso de esta web ha evitado visitas a un centro de urgencias:	si no
9.	Recomanaríeu aquesta web?<br>Recomendaría esta web?	si no
10.	Suggeriments<br>Sugerencias	

Enviar
